# Profiling of gene duplication patterns of sequenced teleost genomes: evidence for rapid lineage-specific genome expansion mediated by recent tandem duplications

**DOI:** 10.1186/1471-2164-13-246

**Published:** 2012-06-15

**Authors:** Jianguo Lu, Eric Peatman, Haibao Tang, Joshua Lewis, Zhanjiang Liu

**Affiliations:** 1 The Fish Molecular Genetics and Biotechnology Laboratory, Aquatic Genomics Unit, Department of Fisheries and Allied Aquacultures and Program of Cell and Molecular Biosciences, Auburn University, Auburn, AL, 36849, USA; 2 J. Craig Venter Institute, 9704 Medical Center Dr., Rockville, MD, 20850, USA; 3 Department of Computer Science and Software Engineering, Auburn University, Auburn, AL, 36832, USA

**Keywords:** Gene duplication, Whole genome duplication, Teleost species, Tandem duplication

## Abstract

**Background:**

Gene duplication has had a major impact on genome evolution. Localized (or tandem) duplication resulting from unequal crossing over and whole genome duplication are believed to be the two dominant mechanisms contributing to vertebrate genome evolution. While much scrutiny has been directed toward discerning patterns indicative of whole-genome duplication events in teleost species, less attention has been paid to the continuous nature of gene duplications and their impact on the size, gene content, functional diversity, and overall architecture of teleost genomes.

**Results:**

Here, using a Markov clustering algorithm directed approach we catalogue and analyze patterns of gene duplication in the four model teleost species with chromosomal coordinates: zebrafish, medaka, stickleback, and *Tetraodon.* Our analyses based on set size, duplication type, synonymous substitution rate (*Ks*), and gene ontology emphasize shared and lineage-specific patterns of genome evolution via gene duplication. Most strikingly, our analyses highlight the extraordinary duplication and retention rate of recent duplicates in zebrafish and their likely role in the structural and functional expansion of the zebrafish genome. We find that the zebrafish genome is remarkable in its large number of duplicated genes, small duplicate set size, biased *Ks* distribution toward minimal mutational divergence, and proportion of tandem and intra-chromosomal duplicates when compared with the other teleost model genomes. The observed gene duplication patterns have played significant roles in shaping the architecture of teleost genomes and appear to have contributed to the recent functional diversification and divergence of important physiological processes in zebrafish.

**Conclusions:**

We have analyzed gene duplication patterns and duplication types among the available teleost genomes and found that a large number of genes were tandemly and intrachromosomally duplicated, suggesting their origin of independent and continuous duplication. This is particularly true for the zebrafish genome. Further analysis of the duplicated gene sets indicated that a significant portion of duplicated genes in the zebrafish genome were of recent, lineage-specific duplication events. Most strikingly, a subset of duplicated genes is enriched among the recently duplicated genes involved in immune or sensory response pathways. Such findings demonstrated the significance of continuous gene duplication as well as that of whole genome duplication in the course of genome evolution.

## Background

Three main mechanisms are believed to generate gene duplications; unequal crossing over, retrotransposition, and chromosomal (genome) duplication [1,2]. Of these, localized (or tandem) duplication resulting from unequal crossing over and genome duplication are believed to be the two dominant mechanisms contributing to vertebrate genome evolution [3,4]. Much energy has been devoted to the examination and modeling of the whole genome duplication events believed to have shaped vertebrate genomes. Over four decades ago, Ohno (1970) suggested that two rounds of large-scale gene duplication had occurred early in vertebrate evolution. Sequencing analysis of Hox gene clusters from a spectrum of vertebrate species provided critical evidence in support of Ohno’s hypothesis [5-8] and indicated, in turn, an additional round of fish-specific genome duplication (FSGD) prior to the divergence of most teleost species [9-13]. Additional evidence supporting FSGD has been garnered from studies of pufferfish, *Takifugu rubripes* and *Tetraodon nigroviridis*. In these studies, hundreds of genes and gene clusters are present in duplicate in teleost fish but possessing only single copy in other vertebrates, illustrating fish-specific duplication of syntenic regions between humans and fish [14-16]. Ongoing examination of gene families across vertebrate evolution continues to provide general support for the three rounds of genome duplication (3R) hypothesis [17-22] in teleost fish.

By contrast, far less energy has been expended in understanding the larger and, arguably, more complicated landscape of gene duplication across model fish genomes and examining how genomes have been shaped and sized by gene duplication forces. Tandem duplication, in particular, is now recognized as a powerful, fast-acting evolutionary mechanism in the generation and expansion of gene families [4], accounting for greater than 10% of human genes [23]. Tandemly-arrayed genes (TAGs) are critical zones of adaptive plasticity, forming the building blocks for sensitive immune, reproductive, and sensory responses [24-26]. However, their extent and impact on teleost genome architecture has been routinely overlooked in the search for broader genome duplication patterns.

While many teleost fish species are in advanced stages of genome sequencing and assembly, only four species currently possess well-annotated genomes with chromosomal-anchored sequence information allowing extensive analysis of gene duplication—zebrafish, *Danio rerio*, medaka, *Oryzias latipes*, green spotted pufferfish, *T. nigroviridis*, and stickleback, *Gasterosteus aculeatus*. These fish, however, represent an interesting cross section of teleost diversity, with genomes differing in size from 342 Mb in pufferfish to 1.5 Gb in zebrafish, and with great variations in effective population sizes and generation intervals ranging from 7 weeks to 2 years. Differences in life history may reasonably be expected to impact patterns of gene duplication and retention. According to the neutral theory of molecular evolution [27] a new paralogous allele, if selectively neutral, has a probability of 1/2 N (where N is effective population size) of being fixed in a diploid population, with fixation occurring, on average, over 4 N generations. Differences in population size and generation interval among the teleost model species may also impact the extent and effectiveness of positive selection as seen previously in comparisons of duplicated genes between human and mouse [28].

Several recent studies have highlighted exceptional features of the zebrafish genome. These include reports of significantly higher rates of evolution in conserved noncoding elements [29], the largest numbers of tandemly-arrayed duplicates among all surveyed vertebrate species [4], and the highest average duplication rate of all lineages in the vertebrate tree (9.04 duplications/Ma [30]). Our own research has previously revealed a potentially related phenomenon of lower levels of alternative splicing when compared to other teleost species [31] and has explored the extensive nature of tandem duplications within some zebrafish gene families, e.g. cc chemokines [32]. Indeed, the particularities of the zebrafish genome have led many studies to use the more canonical pufferfish and medaka genomes in testing genome and gene duplication models and theories. The zebrafish genome may be perceived to represent some of the genome architecture of a large number of vertebrate species given its location on a portion of the tree of life within Cyprinidae with over 2,400 extant species. However, huge diversities exist in this group of freshwater fishes. For instance, the genome of common carp (*Cyprinus carpio*) is believed to have gone through additional round of whole genome duplication. Therefore, in terms of gene duplication, the common carp genome could be drastically different from the architecture of the zebrafish genome. Detailed examination and comparative analysis of the nature and impact of duplications in the zebrafish genome may only provide some reference for gene duplication analysis in related species.

To study the nature and extent of duplication among teleost species, here, we used a Markov clustering dynamic programming algorithm to arrange gene duplicates within the four model fish genomes into sets. Further analyses based on set size, duplication type, synonymous substitution rate (*Ks*), and gene ontology emphasize shared and lineage-specific patterns of genome evolution via duplication. Most strikingly, our analyses confirm the extraordinary duplication and retention rate of recent duplicates in zebrafish and their likely role in the expansion of the zebrafish genome.

## Results

### Duplicated gene sets among four model teleost species

Unigene sets gathered from the Ensembl databases of the four teleost fish were used for self-BLAST (all vs. all) followed by Markov clustering dynamic programming utilizing chromosomal coordinates as implemented in the program MCScan [33]. As shown in Table 1, a total of 3,991, 2,584, 2,669, and 2,020 duplicated gene sets were identified from zebrafish, medaka, stickleback, and green spotted pufferfish (*Tetraodon*), respectively. Based on chromosomal positions and relationships, the duplication sets were divided into three non-exclusive types: tandem duplication, inter-chromosomal duplication (non-tandem) and intra-chromosomal duplication (non-tandem). Definitions for the duplication types were as follows: 1) tandem duplication: duplicated gene copies located within 10 kb of one another (pairwise); 2) Intra-chromosomal duplication (Non-tandem): duplicated gene copies located on the same chromosome with a distance of greater than 10 kb between all members; and 3) Inter-chromosomal duplication (Non-tandem): duplicated gene copies located on different chromosomes. A portion of the duplicated sets combined several duplication types (e.g., duplicate set members present in both tandem and inter-chromosomal arrangements; Table 1). Inter-chromosomal duplications were the most prevalent among the three types across all four teleost species, accounting for around 80% of duplication sets and indicating the importance of genome-level duplication events in shaping teleost genome architecture. Intra-chromosomal and tandem duplication were the second and third most prevalent types, respectively. Zebrafish had the highest percentage of sets within these latter two categories, 47%, compared with 33.9%, 35.5%, and 38.6% in medaka, stickleback, and *Tetraodon*, respectively. In addition, zebrafish differed noticeably from medaka, stickleback, and *Tetraodon* in average duplication set size, with 4.3 genes per duplication set compared to 5.4 genes per set in the three other species.

**Table 1:** Summary of gene duplications in four teleost model species

	**Zebrafish**	**Medaka**	**Stickleback**	***Tetraodon***
**Genes**	26,842	18,027	19,178	14,038
**Duplication sets**	3,991	2,584	2,669	2,020
**Average duplication set size (gene number)**	4.3	5.4	5.4	5.4
**Inter-chromosomal duplication sets**	3,109 (77.9%)	2,249 (87.0%)	2,262 (84.8%)	1,645 (81.4%)
**Intra-chromosomal duplication sets**	1,264 (31.7%)	614 (23.8%)	573 (21.5%)	477 (23.6%)
**Tandem duplication sets**	612 (15.3%)	260 (10.1%)	373 (14.0%)	303 (15.0%)
**Mixed duplication sets**	994	539	539	405

Duplication sets reflect groups of putatively paralogous genes clustered together by MCScan. Duplication type classifications are non-exclusive in some cases (Methods) due to multiple duplication types being found in some sets. The number of these “mixed” type sets is listed below for each species.

### Duplication set size prevalence differs between zebrafish and other teleost species

**Figure 1:**
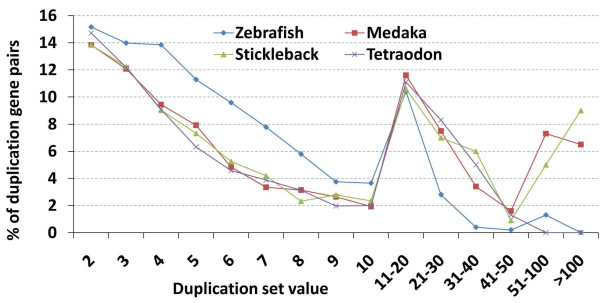
The distribution of duplicated genes from the four model teleost species across varying duplication set sizes

To better understand the distribution of duplicated genes within the four model teleost species, we examined the number of genes on a percentage basis found within duplication sets of varying size. While the relationship between duplication set size and percentage of duplicated genes was similar among the four species (Figure 1; Additional file 1: Table S1.), zebrafish again was the outlier, showing a pattern of more numerous small-scale duplications (set sizes 2–10). This pattern was consistent with our observation of smaller average set size in zebrafish, as was the larger number of duplications found in set sizes greater than 20 in medaka, stickleback, and *Tetraodon*.

### Lineage-specific patterns of duplication events among four teleost species

**Figure 2:**
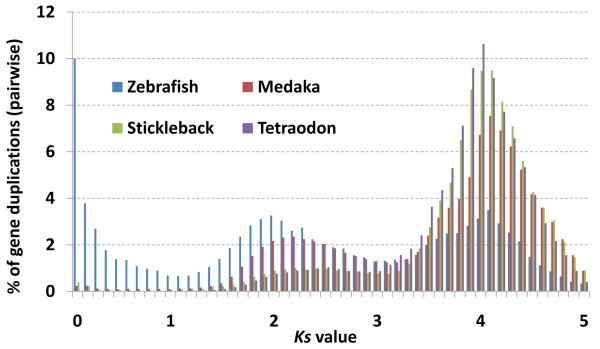
**The distribution of duplicated genes (pairwise comparisons) from the four model teleost species across varying*Ks*values**

We next asked whether the observed prevalence of small duplication sets in zebrafish reflected a faster evolutionary rate in the species as manifested in its duplicated genes. To answer the question, we first examined the mutational distance between the duplicated genes (pairwise) of each species using *Ks*, a measure of the number of substitutions per synonymous site. We again noted a strikingly different *Ks* distribution in zebrafish when compared with the three other model species (Figure 2). Over 24.4% of duplicated genes in zebrafish had *Ks* values of ≤1.0 compared to 1.3%, 0.97%, and 0.05% of duplicated genes in medaka, stickleback and *Tetraodon*, respectively.

**Figure 3:**
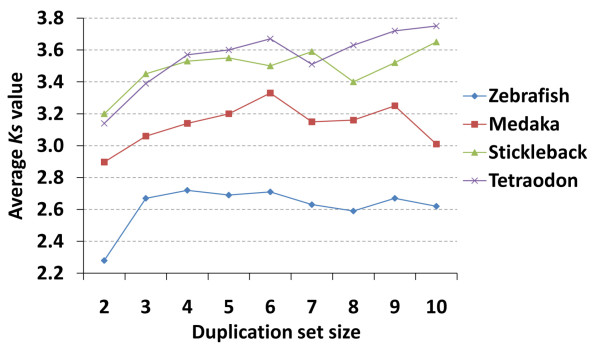
**The relationship between duplication set size and average*Ks*value of duplicated genes from the four teleost species**

To determine whether the abundance of small duplicate sets in zebrafish may be explained by recent evolution (low *Ks*) of these genes, we calculated average *Ks* values for each duplicated set size in the size ranges where zebrafish has a greater percentage of duplicated genes (set size 1–10; Figure 3). Indeed, *Ks* values in these sets are markedly lower in zebrafish than in medaka, stickleback, and *Tetraodon*. Interestingly, while a clear positive correlation existed between duplication set size and *Ks* value in stickleback and *Tetraodon*, this pattern was obscured in medaka and not apparent in zebrafish.

**Figure 4:**
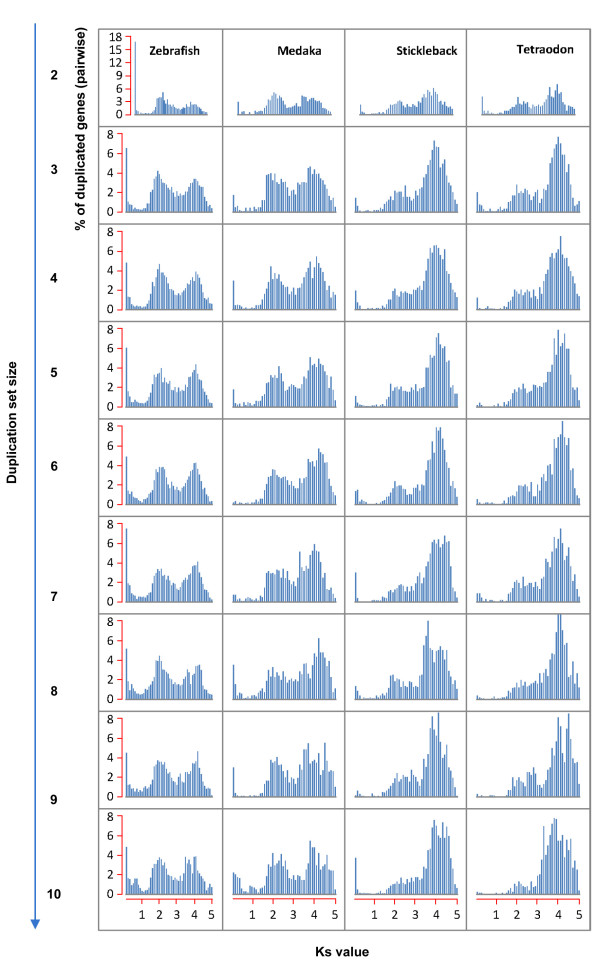
**The distribution of duplicated genes (pairwise comparisons) across increasing*Ks*values for each duplication set size (2 to 10)**

The relationship between *Ks* and set size was even more evident when the duplicated set sizes were analyzed separately and individual pairwise *Ks* values were plotted (Figure 4). As seen previously, zebrafish has an abundance of low *Ks* (*Ks* <1) duplicate pairs at all the studied set sizes when compared with the other three species. However, several other interesting patterns were evident in this analysis. Zebrafish and medaka maintain two roughly proportional peaks of *Ks* values (approximate mean values of *Ks* = 2 and *Ks* = 4), indicating two broad age (divergence level) categories of duplicated genes in these species, irrespective of duplicate set number. In contrast, a single major peak (mean *Ks* = 4) was observed in stickleback and *Tetraodon*, with a much smaller *Ks* peak (*Ks* = 2) appearing to generally diminish with increasing set size. The *Ks* distributions of stickleback and *Tetraodon* are particularly striking in their similarity to one another and suggest a dramatically diminished role for recent duplications in shaping these species’ genomes when compared with zebrafish and medaka.

### Tandem duplications are predominant among small, recent gene duplications in zebrafish

**Figure 5:**
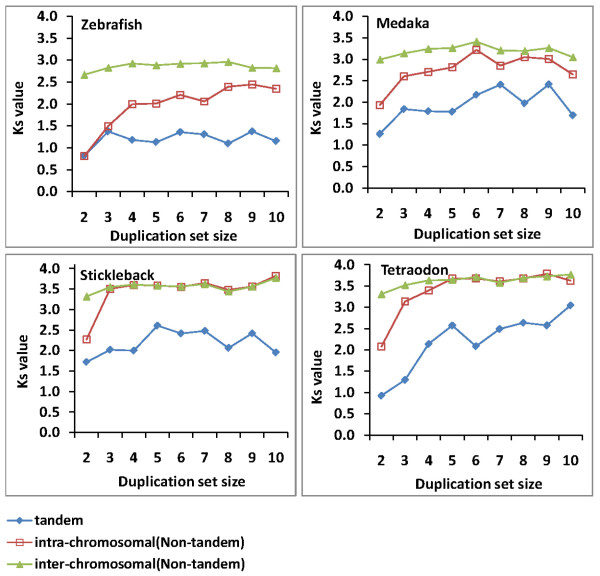
**Average*Ks*values for varying duplication set sizes and among the three different duplication types in the four model teleost species**

We next asked whether the large numbers of small, recent duplications observed in zebrafish were evenly distributed across duplication types or whether they were biased toward a particular type. As seen in Figure 5, tandem gene duplicates had the lowest *Ks* values in each species irrespective of duplication set size. Tandem duplicates from zebrafish had the lowest *Ks* values observed in any species with little perceptible increase in mutational distance across the analyzed duplicated set sizes. Intra-chromosomal duplicates in zebrafish and medaka had intermediate *Ks* values between tandem and inter-chromosomal duplication with an upward trend correlated with increasing duplication set size. By contrast, *Ks* values for intra-chromosomal duplicates in stickleback and *Tetraodon* were virtually indistinguishable from those of inter-chromosomal duplicates in duplication sets of size ≥3. These patterns again point to the static nature of these genomes, with diminished retention and/or minimal levels of recent intra-chromosomal or tandem duplication activity to shape their genome architecture.

### Functional bias of recent (low *Ks*) duplicates in zebrafish

In order to determine whether the expansion of recent, retained duplicates in zebrafish has contributed to the diversification of genes mediating particular physiological functions in the species, we carried out gene ontology analysis on the duplicated gene sets with *Ks* values ≤1.0. This *Ks* range comprises the duplicated set with the most striking expansion when compared with the three other teleost models (Figures 3 and 5). Three GO terms were enriched among these duplicates when compared to the larger set of duplicated zebrafish genes (Additional file 2: Table S2.)—MHC protein complex, olfactory receptor activity, and antigen processing and presentation. Similar enrichment was not detected in the other three species, precluded in part by their small set sizes in this *Ks* range. The enriched categories, critical for immune and sensory capabilities, strongly suggest a functional bias in mechanisms of duplication and retention in zebrafish and further point to the importance of lineage-specific patterns of duplication in genome evolution and species diversification.

## Discussion

Gene duplication has been described as an opportunity to explore forbidden evolutionary space [2], the idea that duplicated genes operating under temporary conditions of relaxed selection provide the raw material for evolution of new gene functions. While whole-genome duplication events are critical in shaping broader genome architecture, gene duplication, particularly tandem events, represent more recent, and potentially, adaptive signatures of evolution [34] which are expected to differ among vertebrate lineages [23,35]. Indeed [36], using zebrafish as their model, and others have shown evidence that evolutionary rates of duplicated genes in teleost fish far outstrip those of the mouse lineage. These differences, aside from adaptive consequences, can have profound effects on the degree of shared ancestry and synteny among vertebrate genomes. For example, only 50% of duplicated genes in zebrafish, and 70% in *Tetraodon*, have their origin in 1R/2R WGD events, compared to over 80% in mammalian, avian, and amphibian lineages. The remaining fraction comes from FSGD and species-specific events [30]. Clearly, patterns of teleost gene duplication deserve closer scrutiny to better understand how this process continues to shape genome evolution. Therefore, here we examined the nature and extent of gene duplication in four model teleosts, zebrafish, medaka, stickleback and *Tetraodon*.

Our approach divided duplicated genes into sets based on duplication type and captured larger gene families as well as smaller, recent duplications. From the onset of our analysis, zebrafish stood out from the other three model species by most measures, with a larger percentage of sets involved in tandem and intra-chromosomal arrangements and numerous small duplication sets (Table 1, Figure 1). Our analysis of the mutational distance between duplicate pairs (*Ks*) across the teleost species (Figure 2), however, produced the most striking illustration of different patterns of duplication and retention. Over 24% of duplicate pairs in zebrafish had *Ks* values of ≤1.0 compared to around 1% or less in the other three species. These results are supported by previous studies which noted high evolutionary rates and duplicate retention rates in zebrafish [29,30]. The abundance of low *Ks* duplicate pairs in zebrafish may stem from a greater number of birth events or fewer gene loss events among young duplicates. Although homogenization through gene conversion is a possibility [2,37,38], the low Ks values are mostly associated with tandem duplicates, suggesting recent gene duplications.

Our approach focused on surveying the broader architecture of duplication in the teleost genomes rather than relying on cross-species phylogenetic analysis for identification of orthologous relationships. Our analyses are limited, therefore, in distinguishing between rapid lineage specific gains in zebrafish and excessive gene loss in other teleosts for particular duplicate sets. The bias in the low *Ks* duplicate pairs in zebrafish toward tandem duplication (Figure 5) provides support for these being recent duplication events. Close to 65% of these zebrafish duplicate pairs with *Ks* ≤ 1.0 are found in tandem arrangements compared with ~15% of total duplicated sets (data not shown). In addition, gene ontology analysis revealed a bias in these duplicates toward physiological functions previously associated with rapid evolution and adaptation [28,39,40]. Indeed, the enriched categories (olfactory receptors, MHC) are well known for their rapid diversification through duplication, recombination, and gene conversion [39,41,42]. Taken together, our results suggest strikingly rapid evolution and high retention of recent duplicates in zebrafish in a manner likely to result in specialization of immune and sensory mechanisms.

The differences observed in *Ks* distributions among the four teleost species (Figures 3 and 5) raised several intriguing questions for further research: What is the effect of life history on the genome architecture of fish, and is there a link between genome size and duplication rate/retention rate in fish? Shiu et al. (2006) examined similar lineage-specific patterns when comparing human and mouse duplicates, suggesting that the larger population size and shorter generation interval in murine species could account for more effective natural selection and retention of duplicated genes. In the four investigated teleost genomes, zebrafish and medaka share similar life history patterns, generation intervals of 7–9 weeks and large effective population sizes, and similar *Ks* distributions (excluding *Ks* <1.0). In contrast, *Tetraodon* and stickleback, with generation intervals of 1–2 year and smaller effective population sizes, had a notable absence of young (low *Ks*) duplicates and shared remarkably similar *Ks* distributions (Figure 5) across their duplicated genes. These patterns of duplication rate and retention have been explored in the light of population size using genome sequence information in invertebrates [43] and previously, on a more theoretical basis [44,45]. Previous observations of correlations between spontaneous duplication/deletion rates and effective population size and increasing retention of linked (tandem) duplicates at intermediate population sizes appear to support such a connection between life history and duplication profiles as suggested by our data. Another pattern deserving further attention as additional teleost genomes become available is a potential association between duplication timing/retention rates and genome size. Based on the limited data available from the four model genomes here, patterns of duplication rate (especially as reflected by those pairs with *Ks* ≤ 1.0) reflect genome size with zebrafish with the largest genome at 1.5 Gb, followed by medaka (700 Mb), stickleback (446 Mb) and *Tetraodon* (342 Mb). The drastically differing patterns of duplicate formation and retention as detected here and by Blomme (2006) may be reflected in evolution of non-coding elements as well [29] and, together, could contribute to significantly higher genic content and associated genome size, as observed in zebrafish [46].

The observed differences in age of duplicated genes as reflected in Ks values could also result from errors in genome sequence assemblies of medaka, stickleback and Tetraodon. As these genomes were sequenced using the shotgun approaches, sequence assembly could have underestimated the segmental duplicated genes. In other words, the most similar paralogues could have been assembled as one gene while they are truly two or more genes in the genome. In this scenario, the missing segmental duplications do affect the assessment of the age of duplications [47]. However, this problem cannot be easily addressed. In order to determine if such a possibility could have caused the major differences in Ks values between zebrafish and the other three fish species, we conducted simulations using zebrafish chromosome 1. The whole genome sequence assembly of zebrafish chromosome 1 was “segmented” into 500 bp pieces and then de novo assembly was conducted using a 10X sequence coverage. In this assembly, a large number of contigs were obtained, 37,396 contigs. Apparently, the large numbers of contigs were resulted from interspersed repetitive segments, most notably the TC1-like transposons. We then mapped the assembled contigs in silico to the reference genome sequence of zebrafish chromosome 1. Over 99.7% of these assembled contigs were mapped to chromosome 1 sequences, suggesting that the “shotgun” approach did not affect the identification of paralogs. Therefore, we believe that the differences in Ks values were likely not caused by sequence assembly errors in medaka, stickleback and Tetraodon although all these genomes were sequenced using whole genome shotgun sequencing.

Previously, we highlighted the low levels of alternative splicing detected from zebrafish (17% of mapped genes) compared with the other model teleost species [31]. By contrast, the compact genome of *Tetraodon* showed alternative splicing in 43% of mapped genes. In that study, an inverse correlation between genome size and alternative splicing was observed. Researchers have previously suggested an inverse relationship between rates of gene duplication and alternative splicing in animals [48] and, more recently, in plants [49] based on single gene or gene family investigations. Our previous analysis of alternative splicing combined with our present examination of gene duplication in the same teleost species appears to support this connection on a genome scale. Further study is warranted to investigate whether the recent duplicates of zebrafish can provide the functional repertoire generated through alternative splicing in other, smaller teleost genomes.

Our findings indicate that varying rates of gene duplication and retention can have a dramatic impact on the ancestry and architecture of teleost genomes and contribute to functional diversification and divergence of important physiological processes. These patterns may be reflective of differences in life history across the teleost radiation and may ultimately influence genic content and genome size. Further analyses of the genomes of additional, key teleosts (i.e. catfish, carp) in the near future will allow us to test these theoretical relationships and analyze the particularities of the zebrafish genome in the context of more recently diverged species.

In Brown’s paper, the Copy number variation elements (CNVE) appeared to be consistent with extensive population substructuring (i.e., local adaption) among zebrafish population, with 4,199 (69%) of the identified CNVEs unique to one strain and only 457 (7.5%) CNVEs are common to all four groups [50]. Given this large amount of genome variation among zebrafish populations, analysis of genomes from additional zebrafish populations may reveal differences in gene copy numbers within a given duplication set. This would be of great interest in helping to establish the rate of gene birth in zebrafish. However, only the reference genome sequences were available for the present analysis. In addition, large differences of gene copy number variations have been mostly associated with anonymous genomic segments, not protein-encoding genes.

## Conclusions

We have analyzed gene duplication patterns and duplication types among the available teleost genomes and found that a large number of genes were tandemly and intrachromosomally duplicated, suggesting their origin of independent and continuous duplication. This is particularly true for the zebrafish genome. Further analysis of the duplicated gene sets indicated that a significant portion of duplicated genes in the zebrafish genome were of recent, lineage-specific duplication events. Most strikingly, a subset of duplicated genes is enriched among the recently duplicated genes involved in immune or sensory response pathways. Such findings demonstrated the significance of continuous gene duplication as well as that of whole genome duplication in the course of genome evolution.

## Methods

### Gene set and duplicated gene search

The zebrafish, medaka, stickleback, and *Tetraodon* protein sequences used in this study were obtained from Ensembl (www.ensembl.org; Ensembl Gene 63; Zv9 for zebrafish, HdrR for medaka, BROAD S1 for stickleback, and TETRAODON 8.0 for *Tetraodon*) were used for the gene duplication analysis. Sequences annotated as unknown, random, and mitochondrial were removed, and only genes with known chromosome location were kept. For all genes with overlapping chromosomal locations, shorter genes were discarded and the longest coding form kept following similar methods used previously [23,34]. Following filtering, there were 26,842 genes in the zebrafish genome, 18,027 genes in the medaka genome, 19,178 genes in the stickleback genome, and 14,038 genes in the *Tetraodon* genome (Table 1). These genes then were used for all-against-all *blastp* searches [51] using the BLOSUM62 matrix and the SEG filter to mask regions of low compositional complexity [52]. Next, all the gene pairs were sorted by gene name and a filter script was used to remove all the redundant pairs, including self matches and multiple matches. These unique and sorted BLAST results were used as the input of MCscan [33]. MCscan is based on a Markov cluster algorithm which retrieves multiple chromosomal regions using dynamic programming based on the similarity matrix generated from previous BLAST results. The default parameter was used (‘mul (0.4343), ceil (200)’) to generate the prerequisite .mcl file for MCscan. For the generated duplication sets, we examined the chromosomal locations of the family members for the following duplication type categories.

### Duplication categories

The copies of the duplicated gene sets may reside on the same chromosome (intra-chromosomal) or on different chromosomes (inter-chromosomal). Based on the locations and arrangements of the duplicated gene copies, we classified the duplicated genes into the following three categories: 1) Tandem duplication: duplicated gene copies are located next to each other on the same chromosome within a distance of less than 10 kb; 2) Intra-chromosomal duplication (Non-tandem): duplicated gene copies are located on the same chromosome with a distance of greater than 10 kb between all set members; and 3) Inter-chromosomal duplication (Non-tandem): duplicated gene copies are located on different chromosomes.

### Synonymous substitution (*K*_*s*_) mutation rates for gene pairs

For each pair of homologs, their protein sequences were aligned with CLUSTALW [53] and their protein alignment converted to DNA alignment with PAL2NAL [54]. The *K*_*s*_ values were calculated using the PAML software package [55]. The Nei-Gojobori algorithm [56] was implemented in the PAML package.

### Gene ontology calculation for gene pairs

Gene ontology enrichment was calculated using goatools [33]. The resulting data structure is based on a directed acyclic graph (DAG) which can be easily traversed from leaf to root. The over-representation and under-representation of certain GO terms were analyzed based on Fisher’s exact test. Also several multiple corrections were implemented including Bonferroni, Sidak, and false discovery rate. The latest version (Jun. 6^th^, 2011) obo-formatted file was downloaded from Gene Ontology website (http://geneontology.org).

### Sequence simulation

The zebrafish chromosome 1 (Zv9) was downloaded from Ensembl database and then it was segmented into 500 bp pieces using CLC bio assembly simulator [57]. De novo assembly was conducted with 10-fold chromosome coverage using CLC Genomics Workbench.

## Competing interests 

The authors declare that they have no competing interests.

## Authors’ contributions

JL conceived the study, carried out the bioinformatics analysis, and was involved in drafting the manuscript. EP conceived the study and was involved in drafting the manuscript. HT conceived the study. JL carried out the bioinformatics analysis. ZL conceived the study, carried out the bioinformatics analysis, and was involved in drafting the manuscript carried out the bioinformatics analysis. All authors read and approved the final manuscript for publication.

## Supplementary Material

Additional file 1 **Table S1.** Duplication set size distribution in four teleost species. Non-bracketed number reflects the number of duplication sets of the listed set size, while the bracketed percentage reflects the percentage of duplicated genes found in the listed set size as represented in Figure 1. Click here for file

Additional file 2 **Table S2.** Gene ontology enrichment in zebrafish duplicate pairs with low *Ks* values (*Ks* ≤ 1.0). Click here for file
