# Supplementary material for: Profiling of gene duplication patterns of sequenced teleost genomes: evidence for rapid lineage-specific genome expansion mediated by recent tandem duplications
Source: BMC Genomics. 2012 Jun 15;13:246. doi: 10.1186/1471-2164-13-246 (PMC3464592; doi:10.1186/1471-2164-13-246)
Supplement: Additional file 1 — Table S1. Duplication set size distribution in four teleost species. Non-bracketed number reflects the number of duplication sets of the listed set size, while the bracketed percentage reflects the percentage of duplicated genes found in the listed set size as represented in Figure 1. [file 1471-2164-13-246-S1.docx]

Supplementary Table 1. Duplication set size distribution in four teleost species. Non-bracketed number reflects the number of duplication sets of the listed set size, while the bracketed percentage reflects the percentage of duplicated genes found in the listed set size as represented in Figure 1.

| **Set size** | **Zebrafish** | **Medaka** | **Stickleback** | ***Tetraodon*** |
| --- | --- | --- | --- | --- |
| **2** | 1309 (15.2%) | 970 (13.8%) | 1005 (13.8%) | 810 (14.7%) |
| **3** | 805 (14.0%) | 564 (12.1%) | 593 (12.2%) | 447 (12.2%) |
| **4** | 598 (13.8%) | 331 (9.4%) | 328 (9.0%) | 250 (9.1%) |
| **5** | 390 (11.3%) | 222 (7.9%) | 213 (7.3%) | 139 (6.3%) |
| **6** | 276 (9.6%) | 113 (4.8%) | 127 (5.2%) | 84 (4.6%) |
| **7** | 192 (7.8%) | 67 (3.3%) | 87 (4.2%) | 61 (3.9%) |
| **8** | 125 (5.8%) | 55 (3.1%) | 42 (2.3%) | 43 (3.1%) |
| **9** | 72 (3.8%) | 41 (2.6%) | 45 (2.8%) | 24 (2.0%) |
| **10** | 63 (3.6%) | 27 (1.9%) | 34 (2.3%) | 22 (2.0%) |
| **11-20** | 134 (10.4%) | 113 (11.6%) | 106 (10.6%) | 84 (11.1%) |
| **21-30** | 20 (2.8%) | 42 (7.5%) | 41 (7.0%) | 37 (8.3%) |
| **31-40** | 2 (0.4%) | 14 (3.4%) | 25 (6.0%) | 16 (5.0%) |
| **41-50** | 1 (0.2%) | 5 (1.6%) | 3 (0.9%) | 3 (1.3%) |
| **51-100** | 4 (1.3%) | 14 (7.3%) | 11 (5.0%) | 0 (0.0%) |
| **>100** | 0 | 6 (6.5%) | 9 (9.0%) | 0 (0.0%) |
