# Supplementary material for: Profiling of gene duplication patterns of sequenced teleost genomes: evidence for rapid lineage-specific genome expansion mediated by recent tandem duplications
Source: BMC Genomics. 2012 Jun 15;13:246. doi: 10.1186/1471-2164-13-246 (PMC3464592; doi:10.1186/1471-2164-13-246)
Supplement: Additional file 2 — Table S2. Gene ontology enrichment in zebrafish duplicate pairs with low Ks values (Ks ≤ 1.0). [file 1471-2164-13-246-S2.docx]

Supplementary Table 2. Gene ontology enrichment in zebrafish duplicate pairs with low *Ks* values (*Ks* ≤ 1.0)

| **GO term** | **GO category** | **GO name** |
| --- | --- | --- |
| GO:0042611 | Cellular component | MHC protein complex |
| GO:0004984 | Molecular function | Olfactory receptor activity |
| GO:0019882 | Biological process | Antigen processing and presentation |
